# Simultaneous upper and lower body perfusion using hypothermia during thoracoabdominal aortic surgery

**DOI:** 10.1186/s13019-023-02439-3

**Published:** 2023-11-14

**Authors:** Yuya Kise, Yukio Kuniyoshi, Mizuki Ando, Keita Miyaishi, Shotaro Higa, Tatuya Maeda, Moriyasu Nakaema, Hitoshi Inafuku, Kojiro Furukawa

**Affiliations:** 1 Department of Thoracic and Cardiovascular Surgery, Graduate School of Medicine, University of the Ryukyus, 207 Uehara, Nishihara, Okinawa 903-0215 Japan; 2 Department of Cardiovascular Surgery, Urasoe General Hospital, Urasoe, Okinawa Japan

**Keywords:** Upper and lower body perfusion, Spinal cord protection, Thoracoabdominal aortic aneurysm, Thoracoabdominal aortic repair, Hypothermia

## Abstract

**Background:**

In open thoracoabdominal aortic aneurysm (TAAA) repair, we have been performing vascular reconstruction under moderate to deep hypothermia and assisted circulation using simultaneous upper and lower body perfusion. This method is effective for protecting the spinal cord and the brain, heart, and abdominal organs and for avoiding lung damage.

**Methods:**

TAAA repair was performed under hypothermia at 20–28 °C in 18 cases (Crawford type I in 0 cases, type II in 5, type III in 3, type IV in 4, and Safi V in 6) between October 2014 and January 2023. Cardiopulmonary bypass was conducted by combined upper and lower body perfusion, with perfusion both via the femoral artery and either transapically or via the descending aorta or the left brachial artery.

**Results:**

The ischemic time for the artery of Adamkiewicz and the main segmental arteries was 40–124 min (75 ± 33 min). No spinal cord ischemic injury or brain or heart complications occurred. One patient with postoperative right renal artery occlusion and one with an infected aneurysm required tracheostomy, but the intubation time for the other 16 was 32 ± 33 h. The duration of postoperative intensive care unit stay was 6.5 ± 6.2 days, the length of hospital stay was 29 ± 15 days, and no in-hospital deaths occurred.

**Conclusions:**

Simultaneous upper and lower body perfusion under moderate to deep hypothermia during thoracoabdominal aortic surgery may avoid not only spinal cord injury, but also cardiac and brain complications.

## Introduction

The frequency of immediate spinal cord ischemic injury (SCII) is higher in open thoracoabdominal aortic aneurysm (TAAA) repair than in thoracic endovascular aortic repair (TEVAR) [1, 2]. The main reason is decreased spinal cord perfusion during aortic cross-clamping. With the aim of prolonging spinal cord ischemic tolerance time during cross-clamping, we use moderate to deep hypothermia and also conduct simultaneous upper and lower body perfusion [3, 4]. The advantages of this method are that it not only maintains the blood supply to the spinal cord via the collateral network and protects the brain and heart, but it also enables the procedure to be conducted without lung damage even when the aortic cross-clamping site is limited due to lung adhesions resulting from chronic dissection or in re-thoracotomy after thoracic descending aortic replacement. The outcomes of 18 patients treated by this method are reported, and its utility is described.

## Patients and methods

### Patients

A total of 18 TAAA patients treated using this method between October 2014 and January 2023 were reviewed. They included 13 men and 5 women of mean age 55 ± 15 years. Marfan syndrome was present in 5 cases. There was no case of Crawford type I, and there were 5 cases of type II, 3 of type III, 4 of type IV, and 6 of Safi V. There were 9 cases of chronic dissection, 5 of true aneurysm, 2 of inflamed aneurysm, and 2 of infected aneurysm. Most of the patients had undergone previous surgery, comprising the modified Bentall (mBentall) procedure or ascending aortic (arch) replacement in 7 cases, thoracic descending aortic replacement in 2, TEVAR in 2, and Y-graft replacement in 4. The artery of Adamkiewicz (AKA) was preoperatively identified in 10 of 18 patients (Table 1). The study was approved by the Ethics Committee of the University of Ryukyus, Japan.

**Table 1:** Patients’ profiles

Case no	Age/Sex	Extent type (etiology)	AKA localization	Previous operations	Comorbidity
1	57/M	II (IIIB CD)	Th11 Lt	TAR, DAR, mBentall, Y graft replacement	
2	66/F	IV (inflammatory)	(–)		
3	50/M	II (IIIB CD)	Th10 Lt	AAR	
4	67/M	III (IIIB CD)	Th10 Lt		
5	65/M	V	(–)		
6	69/M	IV (infectious)	L1 Lt		
7	38/F	II (IIIB CD)	Th12 Rt	mBentall, Y-graft replacement	
8	51/M	IV	Th10 Lt		
9	77/M	V	Th10 Rt	AVR, Y-graft replacement	
10	43/F	V(inflammatory)	(–)	TEVAR	
11	49/M	V	Th8 Rt		
12	46/M	II (IIIB CD)	(–)	mBentall, Y-graft replacement	
13	54/F	III (IIIB CD)	(–)	AAR, DAR	
14	26/M	II (IIIB CD)	(–)	AAR, TAR	
15	57/M	V	(–)		HD
16	74/M	IV (infectious)	Th10 Lt		HD
17	29/M	III (IIIB CD)	Th10 Lt	mBentall, TEVAR	
18	77/F	V (IIIB CD)	(–)		CRD
Average	55 ± 15				

*AKA* Adamkiewicz artery, *CD* chronic aortic dissection, *TAR* total arch replacement, *DAR* descending aortic replacement, *AAR* ascending aortic replacement, *HD* hemodialysis, *CRD* chronic renal disease

### Surgical technique

**Fig. 1:**
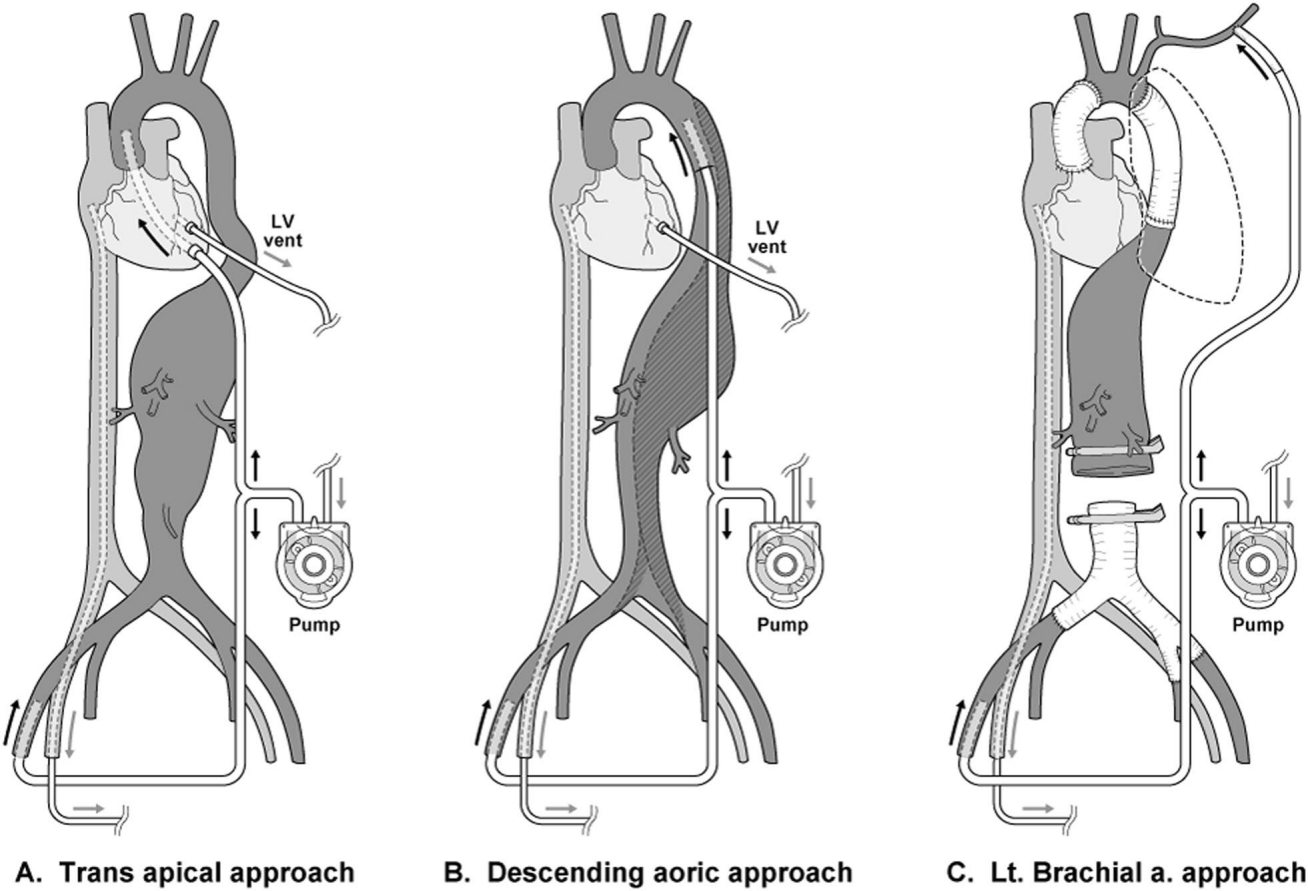
Schema of the simultaneous upper and lower perfusion technique. **A** Transapical aortic and femoral artery perfusion, **B** descending aorta and femoral artery perfusion, **C** left brachial artery and femoral artery perfusion

The general technique has been described previously [3, 4]. After induction of anesthesia, a transcranial motor evoked potential (tcMEP) monitor was fitted, and the control values were measured. Blood pressure monitors were placed in the right radial artery and the left femoral artery. With the patient in the right semi-decubitus position, thoracotomy was conducted by making a Stoney incision in the 5th intercostal space for type II aneurysms, the 7th intercostal space for type III, and the 8th intercostal space for types IV and V, and the diaphragm was dissected in an arc to access the retroperitoneal space. If necessary, one or two anterior and posterior ribs were resected to provide an adequate field of view. For patients with a dissected descending aortic aneurysm or a descending aortic graft that was adhering to the lungs, either Zone III of the pulmonary apex was taped (in 4 cases), or an occlusion catheter (Pruitt®) was used for proximal cross-clamping to avoid unnecessary dissection (in 2 cases). For type III–V aneurysms, taping was carried out immediately above and below the aneurysm to secure the cross-clamping site (in 11 cases). In 1 patient with a type II aneurysm, there was no site in Zone III where cross-clamping was feasible, and an open proximal anastomosis was performed under circulatory arrest. After administration of heparin 300 units/kg, the right femoral artery and vein were cannulated, and cardiopulmonary bypass (CPB) was started. For patients with a type III–V aneurysm and no pulmonary adhesions or atheroma, the thoracic descending aorta was the first choice as the upper body perfusion route. In patients for whom the descending aorta was unsuitable for the perfusion site, this was conducted either transapically in the center of the operating field, or via a perfusion route created by anastomosing a graft prosthesis (7–9 mm bore) to the left brachial artery (recently this method has been preferred), and double perfusion of the upper and lower body was started (Fig. 1). In light of the aortic cross-clamping time and the ischemic time for the important segmental arteries (SAs), central cooling was continued until the bladder and tympanum temperatures reached 20–28 °C. To prevent damage from left ventricular distension before transition to ventricular fibrillation (Vf), a pulmonary artery (PA) vent or a left ventricular (LV) vent from the left ventricular apex was placed (Cases 1–10), but an LV vent was not used when blood drainage was sufficient, and transesophageal echocardiography (TEE) showed that there was no left ventricular distension (Cases 11–18). In patients with mild to moderate aortic regurgitation, their own heartbeats were maintained by gradual cooling to 26–28 °C to prevent left ventricular distension (Cases 15–18). Below 25 °C, tcMEP latency was greatly prolonged, and the amplitude disappeared. After the target body temperature had been achieved by core cooling for 30 min, the taped sites on the proximal and distal sides of the aneurysm were cross-clamped, the aneurysm was resected, and selective perfusion of the abdominal branch was started. In patients with no atheroma of the aneurysm, segmental cross-clamping was conducted as far as possible to minimize the extent of spinal cord ischemia. Upper and lower body perfusion was continued, and continuous infusion of noradrenaline as required was used for control so that upper and lower extremity pressure was maintained at 50–60 mmHg. An Invos® (Mallinckrodt Europe, Hertogenbosch, Netherlands) was used to monitor for any extreme decrease in cerebral perfusion. In patients whose vascular walls were in poor condition and who required some time for AKA reconstruction, the AKA was reconstructed first, and perfusion was carried out from the CPB into the reconstruction graft. In the other cases, the AKA was reconstructed after proximal anastomosis formation. An end-to-side anastomosis was formed between the main graft and the AKA reconstruction graft, the position of the clamp was moved, and antegrade perfusion into the AKA was started, after which the abdominal branch and the distal anastomosis were sequentially reconstructed while the body was being warmed. In patients who had previously undergone Y-graft replacement and those for whom distal cross-clamping was hindered by atheroma, either reconstruction was conducted first by open distal anastomosis (Cases 9 and 12), or an occlusion catheter (Pruitt®) was inserted via the vascular lumen, and distal perfusion was maintained (Cases 15, 16, and 18). The re-appearance of tcMEP amplitude during warming was confirmed. In Case 3 and Case 13, a CSF drainage catheter was placed preoperatively at the discretion of the surgeon when there was a poor intraoperative or postoperative blood supply environment. In both cases, there was no significant increase in CSF pressure intraoperatively or postoperatively.

## Results

Mean operating time was 11.2 ± 3.3 h, extracorporeal circulation (ECC) time was 263 ± 108 min, and minimum body temperature was 22.2 ± 3.8 °C (during the last 3 years it was usually 25–28 °C). The time from aortic cross-clamping to proximal anastomosis was 32–122 min (63 ± 27 min), and the ischemic time for the AKA and the main SAs was 40–124 min (74 ± 29 min) (Tables 2 and 3). No patient developed paraplegia, paraparesis, or other nerve damage due to SCII or cerebral infarction, and no cases of postoperative low output syndrome (LOS) were observed. In terms of serious complications, one patient with right renal artery occlusion (Case 3) and one with an infected aneurysm (Case 6) required postoperative tracheostomy and long-term hospitalization, but they were discharged alive. The intubation time was 32 ± 33 h, the duration of ICU stay was 6.5 ± 6.2 days, and the length of hospital stay was 29 ± 15 days. No postoperative deaths occurred (Table 4). All 18 patients who have undergone the procedure since 2014 have had no complications or rehospitalization due to cardiovascular events after discharge.

**Table 2:** Intraoperative data

Case No	Infusion sites of ECC upper/lower	Vent	Placement of proximal aortic clamp	Clamp time of proximal anastm. (min)	Lowest temperature (°C)	mBP during aortic X clamp upper/lower (mmHg)	ECC flow (ml/kg/min)
1	TA/Rt.FA	PA	Zone III	58	19.3	44–52/52–62	53–68
2	DTA/Rt.FA	LV	Th 9	40	19.1	54–61/58–70	39–56
3	TA/Rt.FA	PA	Zone III	120	18.4	58–58/51–62	47–58
4	DTA/Rt.FA	LV	Th 7	39	18.0	72–92/58–68	39–52
5	DTA/Rt.FA	LV	Th 8	32	19.8	57–84/48–52	51–64
6	DTA/AA	LV	Th 11	41	19.3	42–55/47–74	48–60
7	TA/Rt.FA	LV	Zone III	43	19.4	47–63/50–62	47–58
8	TA/Rt.FA	LV	Th 9	41	20.4	42–56/55–77	37–47
9	Asc.a/Rt.FA	LV	Th 9	83	20.8	42–70/0–35	19–52
10	TA/Rt.FA	LV	Th 11	122	20.1	35–60/26–40	32–58
11	Lt.brac/Rt.FA	(–)	Th 8	44	25.0	45–80/^†^	24–59
12	Lt.brac/Rt.FA	(–)	open proximal	82	19.3	5–90/^†^	15–58
13	Lt.brac/Rt.FA	(–)	Th 8(balloon)	45	25.5	27–90/30–40	12–53
14	Lt.brac/Rt.FA	(–)	Zone III	55	24.0	34–80/^†^	36–78
15	Lt.brac/Rt.FA	(–)	Th11	64*	28.0	60–85/80–100	30–55
16	DTA/Rt.FA	(–)	Th11	88*	28.0	60–70/50–70	42–59
17	Lt.brac/Rt.FA	(–)	Th9^‡^	68*	27.0	55–75/40–100	19–51
18	DTA/Rt.FA	(–)	Th11	72*	28.0	45–80/42–90	24–76
Average				63 ± 27	22.2 ± 3.8		

*TA* Transapical, *DTA* descending thoracic aorta, *LV* left ventricle, *PA* Pulmonary artery, *Asc.a* ascending aorta, *brac* brachial artery

*Distal first anastomosis

^†^No record

^‡^Occlusion balloon

**Table 3:** Intraoperative data

Case no	SA (AKA) reconstruction	Ischemic time of Spinal cord at SA (AKA) reconstruction (min)	Amplitude of MEP monitor	ECC time (min)	Operation time (hours)
1	Th11 Lt, Rt	124	Recovery	285	17
2	(–)	40*	Recovery	201	7.5
3	Th9, Th10 pare	60	Recovery	339	13
4	Th9 Lt, Rt	120	Recovery	337	15.5
5	Th10 Rt	76	Recovery	213	9.5
6	(–)	40*	Recovery	535	15
7	Th12 Rt, L1 Rt	119	Recovery	371	13.5
8	Th10 Beveling	43*	Recovery	194	8
9	Th10,Th11preserving	83*	Recovery	204	9
10	(–)	122*	Recovery	171	6
11	Th8, Th9 preserving	44	Recovery	188	9.5
12	Th11, L1	54	Recovery	460	15.5
13	Th10 preserving	45*	Recovery	184	12
14	Th12, L1	70	Recovery	303	12
15	Th10,Th11 preserving	64	Recovery	142	8.5
16	Th10,Th11 preserving	88	Recovery	236	9.5
17	Th9,Th10 preserving	68	Recovery	218	13.6
18	Th11,Th12 preserving	72	Recovery	166	6.9
Average		74 ± 29		263 ± 108	11.2 ± 3.3

Case no	Blood losses (ml)	Salvaged blood (ml)	RBC (ml)	FFP (ml)	Platelets (units)
1	2042	1400	840	1440	20
2	1327	200	560	480	20
3	4084	1007	1680	1440	30
4	3500	895	740	2400	30
5	1407	915	560	480	20
6	2242	1852	1680	2400	30
7	2053	1200	560	1440	30
8	1706	481	280	1200	20
9	828	967	560	1200	20
10	1470	534	560	0	10
11	1764	244	280	720	20
12	6185	1262	470	1200	30
13	3837	1927	840	1200	20
14	1333	620	840	960	20
15	1560	0	560	960	20
16	965	430	560	960	20
17	4264	1467	840	1680	30
18	2514	923	840	1200	20
Average	2393 ± 1427	906 ± 548	736 ± 386	1186 ± 602	22.8 ± 5.7

**Table 4:** Operative results

Case no.	Intubation time (hours)	ICUstay (days)	Postoperative hospital stay (days)	Spinal cord ischemic injury	Max CKMB (ng/ml)	Complication
1	96	8	24	(–)	153	(–)
2	15	4	24	(–)	187	(–)
3	*	25	40	(–)	261	Rt.RAocclusion, Tracheostomy
4	120	8	28	(–)	463	Brain edema
5	19	4	28	(–)	189	(–)
6	*	20	70	(–)	50	Tracheostomy
7	11	4	21	(–)	70	(–)
8	14	3	22	(–)	114	(–)
9	40	6	26	(–)	46	(–)
10	14	3	22	(–)	53	(–)
11	10	2	13	(–)	32	(–)
12	35	7	41	(–)	8	(–)
13	29	3	31	(–)	28	SSI
14	9	3	18	(–)	56	(–)
15	12	3	16	(–)	32	(–)
16	67	7	60	(–)	46	Sepsis^†^
17	13	4	18	(–)	91	(–)
18	12	3	19	(–)	22	(–)
Average	32 ± 33	6.5 ± 6.2	29 ± 15		105 ± 113	

*SSI* surgical site infection

*Long-term ventilator management after tracheostomy

^†^Bacterial translocation after postoperative 3 weeks

## Comments

The outcomes of open TAAA repair cannot be said to be satisfactory even in experienced centers, and SCII in particular is a critical complication that is characteristic of this procedure [5]. There are numerous factors that hinder the complete avoidance of SCII, including: (1) the presence of numerous fine SAs in the area to be clamped during TAAA repair treated as major vessel reconstruction surgery; (2) despite the importance of intraoperative AKA reconstruction and perfusion, spinal cord protection cannot be completely guaranteed; (3) due to an abundant spinal cord collateral network, stealing from the SAs within the area of aortic cross-clamping causes a major decrease in spinal cord perfusion pressure; (4) similar to the brain, the spinal cord, which is part of the nervous system, has a very short ischemic tolerance time; and (5) intraoperative spinal cord blood supply circulation is not easily evaluated. Overcoming these issues and avoiding intraoperative SCII, that is, immediate SCII, is crucial, and many institutions have reported the measures they take to achieve this.

Safi et al. achieved good results (SCII incidence 4%) by using the left heart bypass method at normal body temperature to mild hypothermia and inserting occlusion catheters into multiple SAs to be preserved (mainly Th8–12) to prevent stealing while conducting reconstruction in a short time by the inclusion technique [6]. Ets et al. conducted preoperative coil embolization of the SAs within the reconstruction area, spread over several procedures, as preconditioning of the preoperative spinal cord blood supply circulation. Their aim was to encourage collateral network development by the gradual elimination of antegrade blood flow to the spinal cord from the SAs and achieve ischemic tolerance of the spinal cord in preparation for open repair (SCII incidence 1%) [7].

Only a few high-volume centers are capable of performing Safi et al.’s sophisticated procedure with good results. Ets et al.’s strategy will also require time to become generally used.

The most important reason for our use of hypothermia as the first-choice method is to prolong spinal cord ischemic tolerance time. At 37 °C, spinal cord infarction generally occurs after 20 min, but at 28 °C this is extended to 75 min and at 20 °C to 120 min [8]. This offers advantages, including: (1) AKA reconstruction can be performed carefully and thoroughly, rather than in a hurry; (2) even if the AKA or important SAs are present between the sites of cross-clamping and anastomosis, they can be left untouched, and vascular reconstruction can proceed while stealing via back flow from collateral routes (allowing spinal cord perfusion pressure to decrease) is tolerated [mean spinal cord ischemic time was 74 min (40–124 min) in the present study, but no spinal cord injury occurred]; and (3) extensive cross-clamping can be conducted with peace of mind even in cases in which segmental cross-clamping is difficult due to atheroma and/or calcification of the vessel wall, such as patients with a shaggy aorta.

When hypothermia is used, the proximal anastomosis is normally created under circulatory arrest [9, 10], but we conduct simultaneous upper and lower body perfusion [3, 4]. One reason is the concern that, under circulatory arrest, air and/or atheroma in the proximal anastomosis may embolize to the brain, an occurrence with a reported incidence of 4–9%. The incidence of postoperative LOS due to myocardial perfusion failure is 1.2–14%, a figure that is also not negligible [11–13]. Maintaining upper body perfusion enables cardiac and cerebral complications to be avoided, and none of the present patients who underwent this procedure suffered stroke or LOS. In Case 4, however, delayed arousal due to postoperative cerebral edema was observed. The upper extremity blood pressure during aortic cross-clamping was high, and hyperperfusion may have occurred, suggesting that caution is required concerning the distribution of blood to the upper and lower body. Another advantage is that upper body perfusion maintains the blood supply to the spinal cord from collateral routes via the vertebrobasilar artery and the internal thoracic artery, among others [14].

In patients undergoing reconstruction procedure after descending aortic replacement and those with chronic dissection causing strong adhesions to the lungs, temporary circulatory arrest was used, and after the aneurysm has been opened, upper body perfusion was maintained by the insertion of an occlusion catheter from the vascular lumen, which enabled minimal lung dissection to be conducted and the anastomosis opening to be formed in a bloodless field of view (Cases 13 and 17). A similar procedure also enabled dissection to be minimized in patients who had previously undergone Y-graft replacement and/or who had strong adhesions around the distal anastomosis site (Cases 15, 16, and 18). In all cases, this procedure was conducted under hypothermia, and it could be safely carried out as low flow in perfusion from the ECC is tolerable. During Vf transition with cooling, the patient should be carefully monitored for left ventricular distension using TEE. In this case, blood drainage must be sped up, or an LV vent must be added. Caution is required for patients with mild to moderate aortic regurgitation, since these patients may develop left ventricular distension as a result of Vf transition during cooling. In these patients, their own heartbeats were preserved by carrying out gradual cooling to 26–28 °C (with the heart rate slowed to 40–60 bpm) (Cases 15, 16, 17, and 18).

Hypothermia has many advantages in TAAA repair, but the reports in the literature of issues, including a tendency to bleeding and lung damage, also warrant attention [9, 13]. Both ensuring hemostasis of the dissected surfaces (including the intercostal thoracotomy, diaphragm, and retroperitoneum) prior to ECC (dry in) and thorough hemostasis after the end of ECC (dry out) are important. To avoid lung damage (pulmonary hemorrhage), every effort should be made to minimize traction on the lungs and to carry it out with care when extending the intraoperative field of view.

## Conclusion

The use of moderate to deep hyperthermia and simultaneous upper and lower body perfusion during TAAA repair may help avoid not only spinal cord injury, but also cardiac and cerebral complications.

## Data Availability

Not applicable.

## Abbreviations

TAAA: Thoracoabdominal aortic aneurysm
SCII: Spinal cord ischemic injury
AKA: The artery of Adamkiewicz
SAs: Segmental arteries
tcMEP: Transcranial motor evoked potential
CPB: Cardiopulmonary bypass
FA: Femoral artery
TEVAR: Thoracic endovascular aortic repair
